# Supplementary material for: Chromosome-level genome assemblies of two parasitoid biocontrol wasps reveal the parthenogenesis mechanism and an associated novel virus
Source: BMC Genomics. 2023 Aug 5;24:440. doi: 10.1186/s12864-023-09538-4 (PMC10403939; doi:10.1186/s12864-023-09538-4)
Supplement: Supplementary file 1 — Additional file 1. [file 12864_2023_9538_MOESM1_ESM.pdf]

Supporting information for

Chromosome-level genome assemblies of two parasitoid biocontrol wasps reveal the parthenogenesis mechanism and an associated novel virus.

Inwood, S.N.[1]§, Skelly, J.[1,2]§, Guhlin, J.G.[3], Harrop, T.W.R.[4], Goldson, S.L.[5], Dearden, P.K.[1,3]*

[1] Bioprotection Aotearoa and Biochemistry Department, University of Otago, Dunedin, Aotearoa-New Zealand
[2] Humble Bee Bio, Wellington, Aotearoa-New Zealand
[3] Genomics Aotearoa, University of Otago, Dunedin, Aotearoa-New Zealand.
[4] Melbourne Bioinformatics, The University of Melbourne, Parkville, VIC, 3010, Australia
[5] Biocontrol and Biosecurity Group, AgResearch Limited, Lincoln, Aotearoa-New Zealand.

*To whom correspondence should be addressed.
§ These first authors contributed equally to this work and are listed alphabetically.

This file contains:
Supplementary figures 1-5
Supplementary tables 1-6

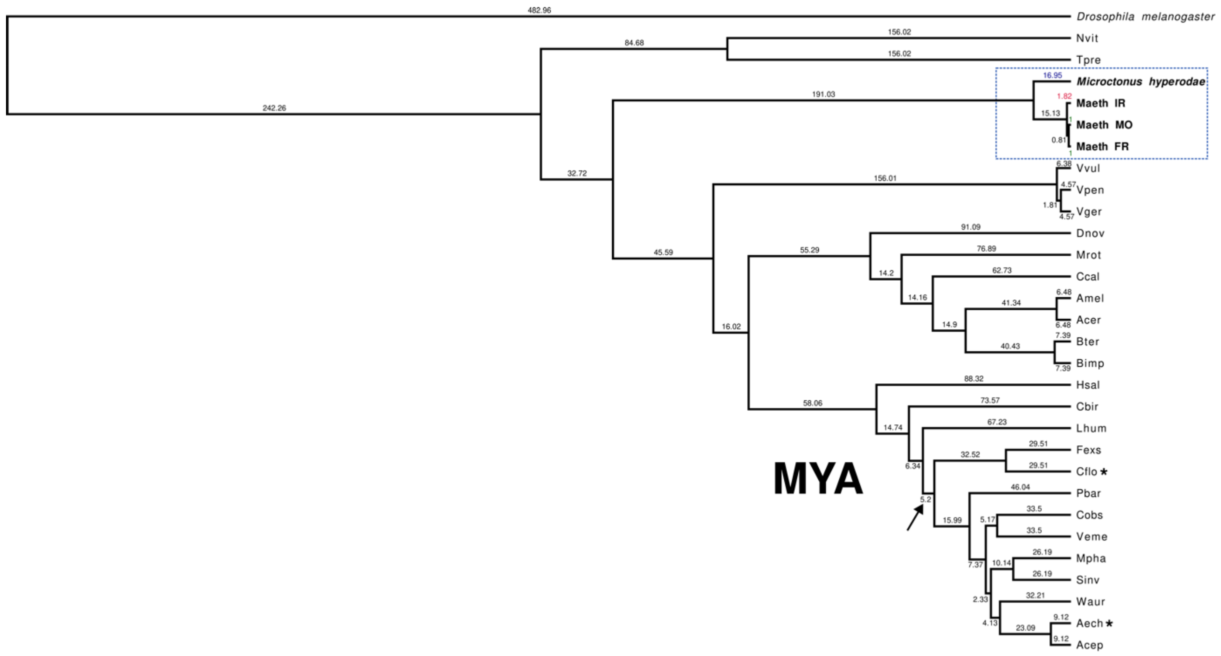

Supplementary Figure 1 Rudimentary estimation of *Microctonus* evolutionary origin based on an ultrametric Orthofinder species tree, using a branch length scaling factor of 563.12. The *Microctonus* clade is highlighted in the blue dashed box. The “Irish”, “French” and “Moroccan” strains of *Microctonus aethiopoides* are Maeth IR, Maeth FR and Maeth MO respectively. *Drosophila melanogaster* is the outgroup. The divergence of *Acromyrmex echinatior* (*) and *Camponotus floridanus* (*) from Peters et al. (2017) was used to calibrate the molecular clock and is indicated by an arrow. The Hymenopteptera abbreviations are as follows: Nvit (*Nasonia vitripennis*), Tpre (*Trichogramma pretiosum*), Vvul (*Vespula vulgaris*), Vpen (*Vespula pensylvanica*), Vger (*Vespula germanica*), Dnov (*Dufourea novaeangliae*), Mrot (*Megachile rotundata*), Ccal (*Ceratina calcarata*), Amel (*Apis mellifera*), Acer (*Apis cerana*), Bter (*Bombus terrestris*), Bimp (*Bombus impatiens*), Hsal (*Harpegnathos saltator*), Cbir (*Cerapachys biroi*), Lhum (*Linepithema humile*), Fexs (*Formica exsecta*), Cflo (*Camponotus floridanus*), Pbar (*Pogonomyrmex barbatus*), Cobs (*Cardiocondyla obscurior*), Veme (*Vollenhovia emeryi*), Mpha (*Monomorium pharaonis*), Sinv (*Solenopsis invicta*), Waur (*Wasmannia auropunctata*), Aech (*Acromyrmex echinatior*) and Acep (*Atta cephalotes*).

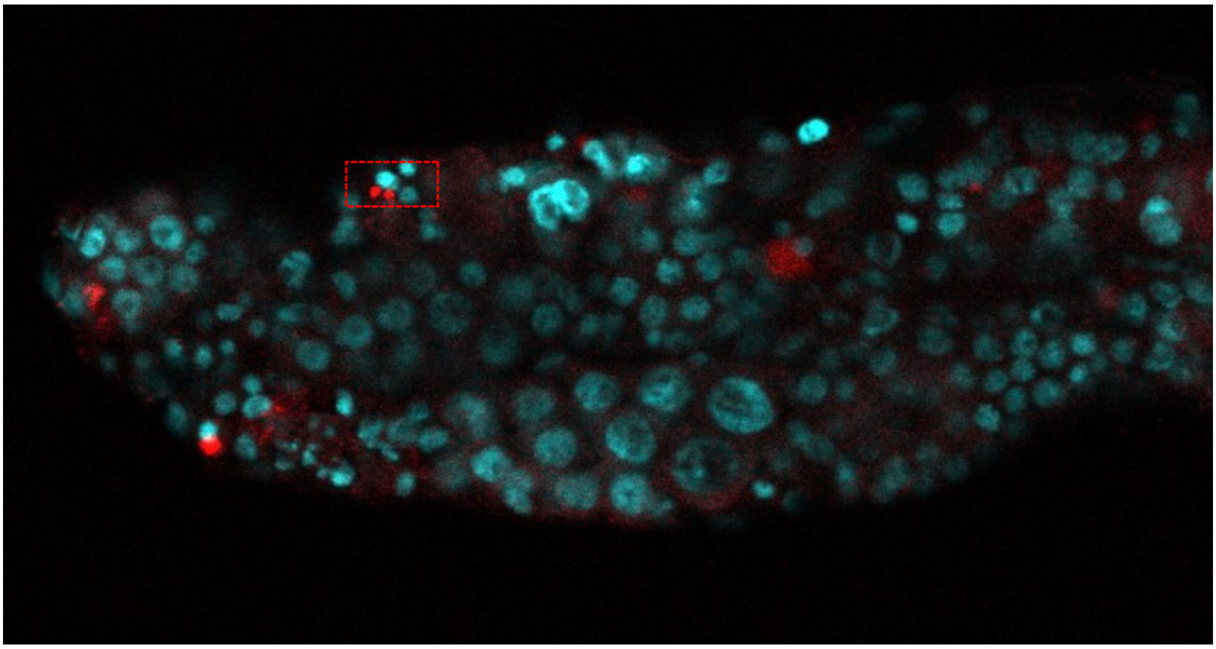

Supplementary Figure 2 HCR in situ hybridisation of MND1 (red) and DAPI staining (cyan) in the ovaries of asexual “Irish” *M. aethiopoides,* with MND1 expression highlighted by the dashed red box.

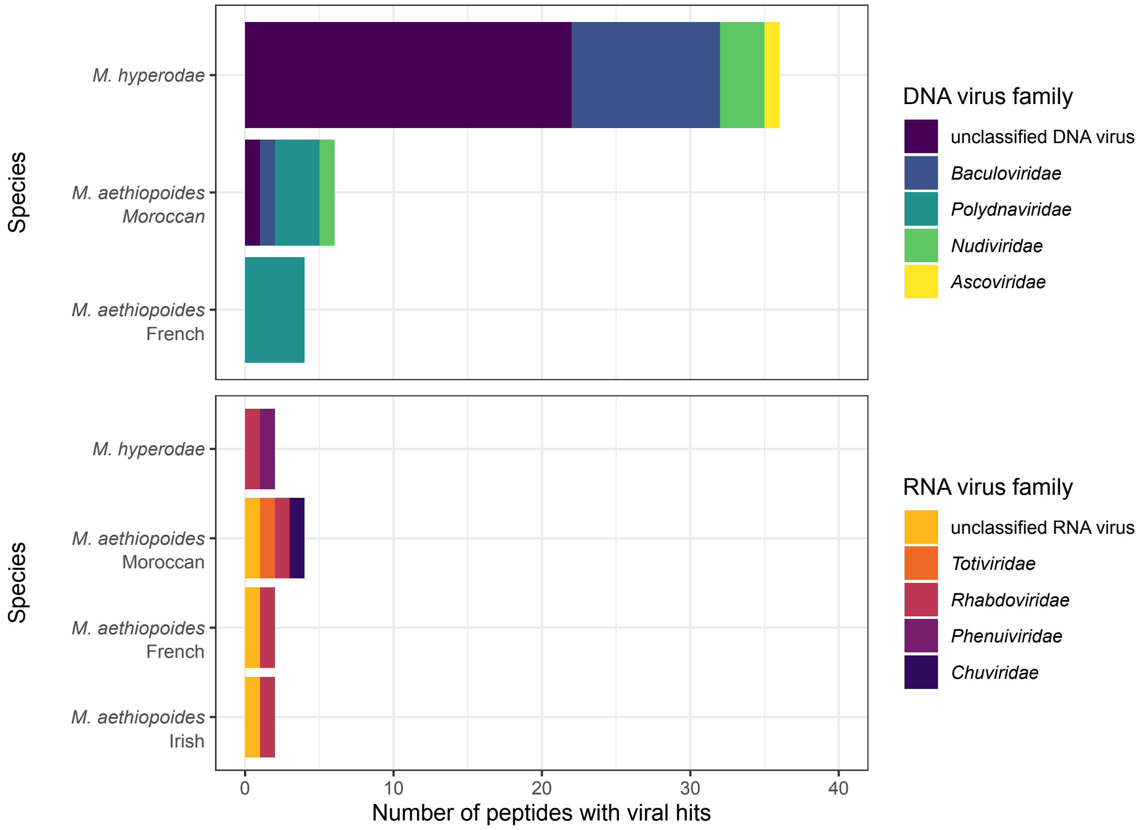

Supplementary Figure 3 Stacked bar graphs showing the number of significant BlastP hits to viral families for predicted proteins from *Microctonus* genome assemblies. Significant BlastP hits were those with E-values below the E-value threshold of 1E-05. Viral hits are divided into two panels, the upper containing hits to DNA viruses, and the lower to RNA viruses. *M. aethiopoides* Irish is not shown in the upper panel due to having no DNA virus hits.

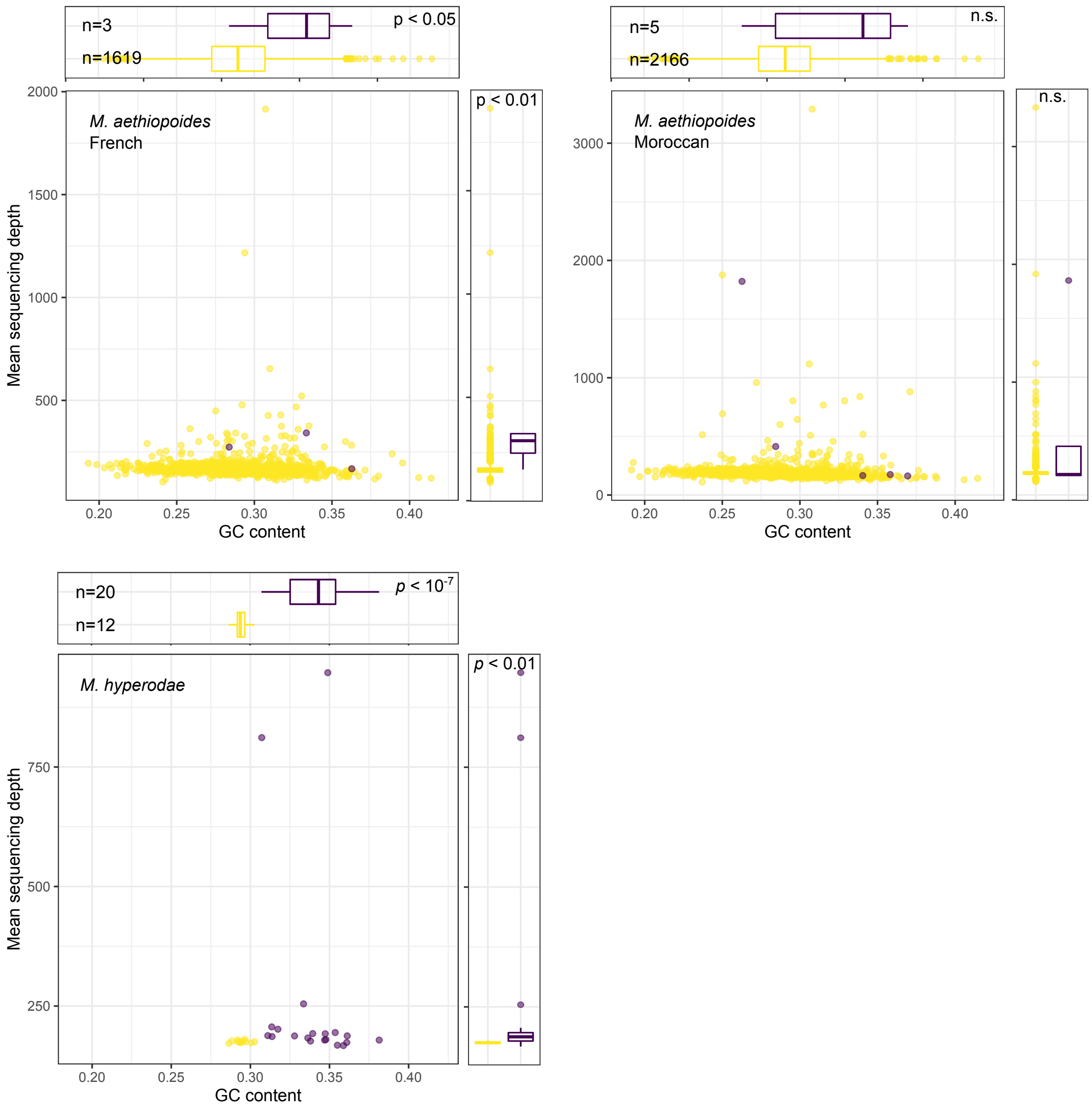

Supplementary Figure 4 Scatterplots and boxplots of mean sequencing depth and GC content of contigs in *Microctonus* genomes. Contigs with viral genes are in purple, and contigs with BUSCO orthologs (for *M. aethiopoides* strains) or Hi-C scaffolds (for *M. hyperodae*), in yellow. Reported P-values were determined using a Kruskal-Wallis test, followed by a pairwise Wilcox test. The number of contigs in each group is reported in the GC content boxplot.

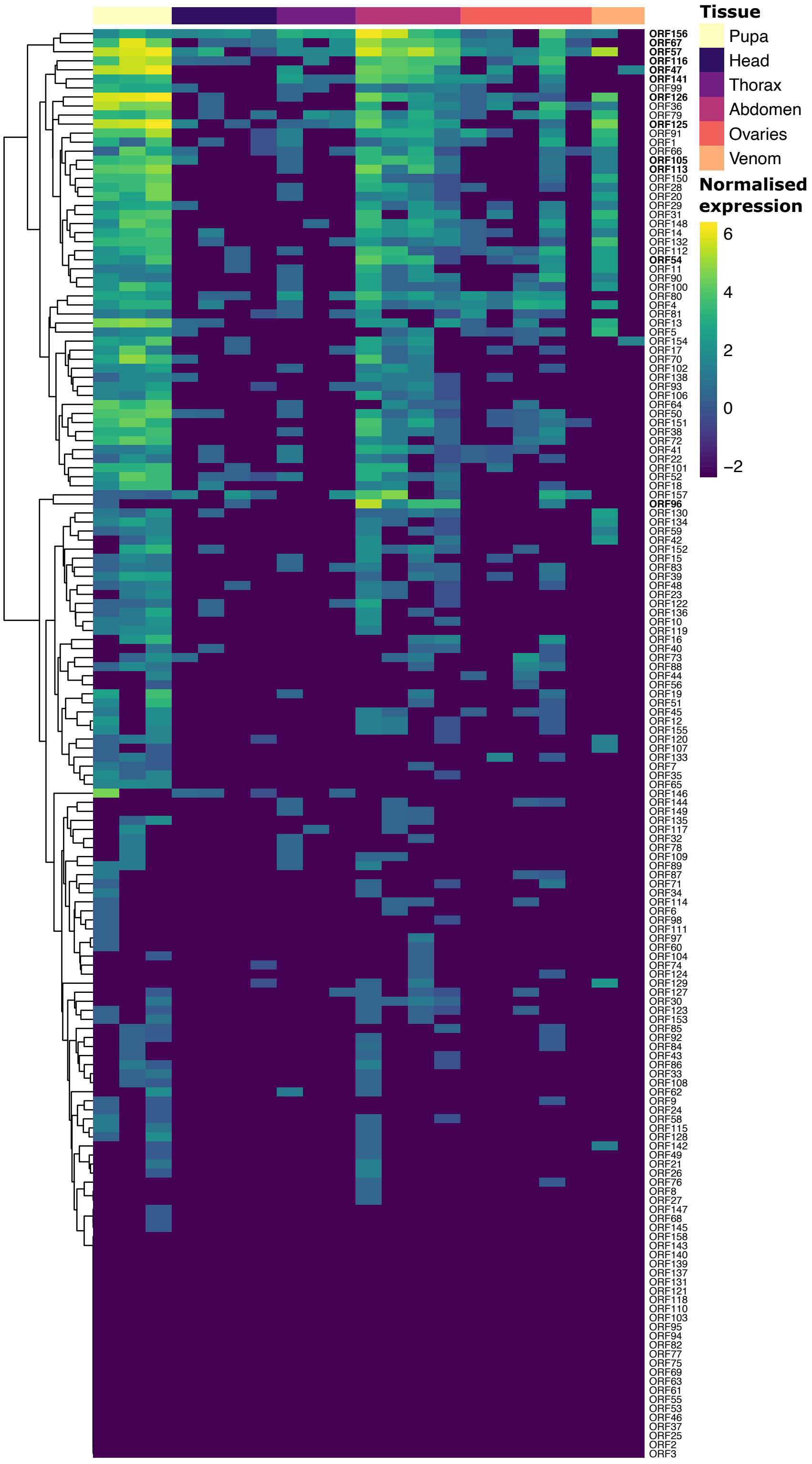

Supplementary Figure 5 A clustered heatmap showing expression for all MhFV genes, across the pupa, head, thorax, abdomen, ovaries and venom of *M. hyperodae*, normalized by VST. Genes detected as significantly differentially expressed in the tissue LRT analysis are indicated in bold.

Supplementary Table 1 Viral hits in *Microctonus* genomes from the reciprocal BlastP analysis.

Assembly	Protein ID	Nr database ID	Seq ID (%)	Hit alignment length	No. of mismatches	E-value	Virus Genome Type	Virus family	BlastP Hit Annotation
M. aethiopoides French	MaethFR_002791-T1	YP_009333371.1	32.48	431	240	3.18E-65	RNA	unclassified RNA virus	putative capsid(Beihai barnacle virus 12)
M. aethiopoides French	MaethFR_006014-T1	YP_184879.1	53.02	149	40	5.76E-38	DNA	Polydnaviridae	hypothetical protein CcBV_30.5 (Cotesia congregata bracovirus)
M. aethiopoides French	MaethFR_006770-T1	YP_184879.1	55.71	149	36	8.36E-40	DNA	Polydnaviridae	hypothetical protein CcBV_30.5 (Cotesia congregata bracovirus)
M. aethiopoides French	MaethFR_007247-T1	QMP82140.1	32.81	384	244	3.53E-56	RNA	Rhabdoviridae	nucleocapsid protein (Hymenopteran rhabdo-related virus OKIAV109)
M. aethiopoides French	MaethFR_012781-T1	YP_184879.1	65.49	113	38	1.17E-37	DNA	Polydnaviridae	hypothetical protein CcBV_30.5 (Cotesia congregata bracovirus)
M. aethiopoides French	MaethFR_013238-T1	YP_009665774.1	28.30	159	81	9.55E-08	DNA	Polydnaviridae	putative recombinase (Chelonus inanitus bracovirus)
M. aethiopoides Irish	MAIEPGA_003765-T1	YP_009333371.1	32.48	431	240	3.18E-65	RNA	unclassified RNA virus	putative capsid (Beihai barnacle virus 12)
M. aethiopoides Irish	MAIEPGA_011016-T1	QMP82140.1	33.07	384	243	1.08E-57	RNA	Rhabdoviridae	nucleocapsid protein (Hymenopteran rhabdo-related virus OKIAV109)
M. aethiopoides Moroccan	MaethMA_001154-T1	YP_009553381.1	38.55	179	105	1.07E-29	DNA	Nudiviridae	OrNV gp005-like protein (Tomelloso virus)
M. aethiopoides Moroccan	MaethMA_003169-T1	YP_009513098.1	28.70	223	124	1.20E-06	DNA	Baculoviridae	pif-0 (Agrotis segetum granulovirus)
M. aethiopoides Moroccan	MaethMA_003175-T1	QKE44565.1	28.19	227	132	7.13E-09	DNA	unclassified DNA virus	hypothetical protein Yalta_118 (Yalta virus)
M. aethiopoides Moroccan	MaethMA_004063-T1	QMP82140.1	32.38	383	247	5.97E-58	RNA	Rhabdoviridae	nucleocapsid protein (Hymenopteran rhabdo-related virus OKIAV109)
M. aethiopoides Moroccan	MaethMA_006925-T1	YP_184879.1	53.02	149	40	5.76E-38	DNA	Polydnaviridae	hypothetical protein CcBV_30.5 (Cotesia congregata bracovirus)

M. aethiopoides Moroccan	MaethMA_009823-T1	YP_009665774.1	28.48	151	75	1.98E-06	DNA	Polydnaviridae	putative recombinase (Chelonus inanitus bracovirus)
M. aethiopoides Moroccan	MaethMA_012135-T1	QPL15382.1	42.99	107	57	7.94E-22	RNA	Chuviridae	glycoprotein (Hymenopteran chu-related virus OKIAV146)
M. aethiopoides Moroccan	MaethMA_012769-T1	YP_009230207.1	27.66	535	350	8.44E-41	RNA	Totiviridae	capsid protein (Camponotus nipponicus virus)
M. aethiopoides Moroccan	MaethMA_012969-T1	YP_009333371.1	32.72	431	239	8.90E-66	RNA	unclassified RNA virus	putative capsid (Beihai barnacle virus 12)
M. aethiopoides Moroccan	MaethMA_013149-T1	YP_184879.1	36.96	138	68	6.36E-13	DNA	Polydnaviridae	hypothetical protein CcBV_30.5 (Cotesia congregata bracovirus)
M. hyperodae	MHPGA_002739-T1	AYD68237.1	45.71	280	136	4.36E-78	DNA	Ascoviridae	bro15 (Heliothis virescens ascovirus 3h)
M. hyperodae	MHPGA_010088-T1	QDH05992.1	35.19	233	143	6.86E-31	DNA	Baculoviridae	Ld-bro-m (Lymantria dispar multiple nucleopolyhedrovirus)
M. hyperodae	MHPGA_010090-T1	YP_009121838.1	54.55	77	31	2.86E-18	DNA	Baculoviridae	bro (Spodoptera frugiperda granulovirus)
M. hyperodae	MHPGA_005949-T1	YP_001649041.1	59.26	54	22	4.64E-13	DNA	Baculoviridae	hypothetical protein HaGV_gp059 (Helicoverpa armigera granulovirus)
M. hyperodae	MHPGA_004603-T1	AOL57072.1	47.59	435	218	3.84E-137	DNA	Baculoviridae	BRO-B (Chrysodeixis includens nucleopolyhedrovirus)
M. hyperodae	MHPGA_004632-T1	AOL56505.1	45.34	483	231	7.85E-138	DNA	Baculoviridae	BRO-B (Chrysodeixis includens nucleopolyhedrovirus)
M. hyperodae	MHPGA_005874-T1	AOL57072.1	44.57	359	151	1.45E-99	DNA	Baculoviridae	BRO-B (Chrysodeixis includens nucleopolyhedrovirus)
M. hyperodae	MHPGA_005950-T1	YP_009116982.1	44.60	444	234	8.13E-133	DNA	Baculoviridae	baculovirus repeat ORF (Pseudoplusia includens SNPV IE)
M. hyperodae	MHPGA_002161-T1	YP_009506121.1	27.98	193	113	1.30E-12	DNA	Baculoviridae	hypothetical protein (Trichoplusia ni granulovirus LBIV-12)
M. hyperodae	MHPGA_005965-T1	YP_009506218.1	48.39	279	128	1.03E-86	DNA	Baculoviridae	bro (Trichoplusia ni granulovirus LBIV-12)
M. hyperodae	MHPGA_004634-T1	AXU41742.1	39.31	145	84	7.73E-22	DNA	Baculoviridae	BRO-B (Spodoptera eridania nucleopolyhedrovirus)

M. hyperodae	MHPGA_011579-T1	ABF93346.1	39.39	132	77	3.44E-22	DNA	Nudiviridae	unknown (Oryctes rhinoceros nudivirus)
M. hyperodae	MHPGA_011580-T1	YP_009553381.1	42.53	87	47	1.17E-10	DNA	Nudiviridae	OrNV gp005-like protein (Tomelloso virus)
M. hyperodae	MHPGA_011609-T1	YP_009553381.1	37.09	151	91	1.83E-21	DNA	Nudiviridae	OrNV gp005-like protein (Tomelloso virus)
M. hyperodae	MHPGA_002066-T1	YP_009305145.1	33.33	258	164	2.89E-36	RNA	Phenuiviridae	nucleocapsid protein (Yichang Insect virus)
M. hyperodae	MHPGA_010179-T1	QMP82140.1	30.65	385	212	3.81E-48	RNA	Rhabdoviridae	nucleocapsid protein (Hymenopteran rhabdo-related virus OKIAV109)
M. hyperodae	MHPGA_002155-T1	YP_009345687.1	24.94	441	264	2.38E-13	DNA	unclassified DNA virus	hypothetical protein LbFV_ORF83 (Leptopilina boulardi filamentous virus)
M. hyperodae	MHPGA_002156-T1	YP_009345706.1	35.48	155	83	6.72E-16	DNA	unclassified DNA virus	hypothetical protein LbFV_ORF102 (Leptopilina boulardi filamentous virus)
M. hyperodae	MHPGA_002164-T1	YP_009345671.1	36.79	280	163	3.29E-46	DNA	unclassified DNA virus	hypothetical protein LbFV_ORF67 (Leptopilina boulardi filamentous virus)
M. hyperodae	MHPGA_003033-T1	YP_009345606.1	31.75	337	199	3.55E-37	DNA	unclassified DNA virus	hypothetical protein LbFV_ORF2 (Leptopilina boulardi filamentous virus)
M. hyperodae	MHPGA_003111-T1	YP_009345691.1	37.36	174	80	7.17E-19	DNA	unclassified DNA virus	hypothetical protein LbFV_ORF87 (Leptopilina boulardi filamentous virus)
M. hyperodae	MHPGA_003113-T1	YP_009345623.1	40.11	177	102	9.18E-30	DNA	unclassified DNA virus	hypothetical protein LbFV_ORF19 (Leptopilina boulardi filamentous virus)
M. hyperodae	MHPGA_003205-T1	YP_009345662.1	37.80	1307	732	0	DNA	unclassified DNA virus	putative DNA pol (Leptopilina boulardi filamentous virus)
M. hyperodae	MHPGA_004604-T1	YP_009345656.1	34.08	267	151	3.10E-30	DNA	unclassified DNA virus	hypothetical protein LbFV_ORF52 (Leptopilina boulardi filamentous virus)
M. hyperodae	MHPGA_004607-T1	YP_009345617.1	28.70	324	200	2.23E-23	DNA	unclassified DNA virus	JmJC domain protein (Leptopilina boulardi filamentous virus)
M. hyperodae	MHPGA_004610-T1	YP_009345647.1	25.93	270	169	6.85E-09	DNA	unclassified DNA virus	hypothetical protein LbFV_ORF43 (Leptopilina boulardi filamentous virus)
M. hyperodae	MHPGA_004649-T1	YP_009345696.1	37.73	599	343	1.52E-113	DNA	unclassified DNA virus	hypothetical protein LbFV_ORF92 (Leptopilina boulardi filamentous virus)
M. hyperodae	MHPGA_004650-T1	YP_009345696.1	25.67	927	529	1.03E-54	DNA	unclassified DNA virus	hypothetical protein LbFV_ORF92 (Leptopilina boulardi filamentous virus)
M. hyperodae	MHPGA_006960-T1	YP_009345700.1	37.85	1025	518	0	DNA	unclassified DNA virus	hypothetical protein LbFV_ORF96 (Leptopilina boulardi filamentous virus)

M. hyperodae	MHPGA_008127-T1	YP_009345624.1	33.52	355	209	6.83E-52	DNA	unclassified DNA virus	hypothetical protein LbFV_ORF20 (Leptopilina boulardi filamentous virus)
M. hyperodae	MHPGA_009336-T1	YP_009345682.1	41.88	640	313	1.98E-135	DNA	unclassified DNA virus	hypothetical protein LbFV_ORF78 (Leptopilina boulardi filamentous virus)
M. hyperodae	MHPGA_009338-T1	YP_009345672.1	32.65	634	373	4.03E-101	DNA	unclassified DNA virus	hypothetical protein LbFV_ORF68 (Leptopilina boulardi filamentous virus)
M. hyperodae	MHPGA_010089-T1	YP_009345667.1	38.51	657	341	5.85E-136	DNA	unclassified DNA virus	hypothetical protein LbFV_ORF63 (Leptopilina boulardi filamentous virus)
M. hyperodae	MHPGA_011597-T1	YP_009345710.1	40.33	600	340	2.39E-144	DNA	unclassified DNA virus	putative ODV protein (Leptopilina boulardi filamentous virus)
M. hyperodae	MHPGA_003110-T1	QKN22504.1	28.81	361	200	3.63E-38	DNA	unclassified DNA virus	putative lecithine cholesterol acyltransferase (Drosophila-associated filamentous virus)
M. hyperodae	MHPGA_004609-T1	QKN22505.1	46.37	289	148	1.61E-82	DNA	unclassified DNA virus	putative protein 51 (Drosophila-associated filamentous virus)
M. hyperodae	MHPGA_004631-T1	QKN22485.1	29.23	349	177	4.55E-20	DNA	unclassified DNA virus	putative ORF5 (Drosophila-associated filamentous virus)
M. hyperodae	MHPGA_011596-T1	QKN22506.1	24.21	504	306	1.98E-22	DNA	unclassified DNA virus	PIF1-like protein (Drosophila-associated filamentous virus)

Supplementary Table 2 A comparison of MhFV virus genome characteristics to other nuclear arthropod-specific large double-stranded DNA viruses.

Virus name	Family	Assembly accession	Genome size (bp)	# genes	GC content	Coding density	Number of MhFV genes with BlastP hits to species	Number of MhFV genes with best BlastP hit to species
Heliothis virescens ascovirus 3f	Ascoviridae	AF451898	198,157	190	45.5	79.7	20	0
Spodoptera frugiperda ascovirus 1a	Ascoviridae	NC_008361.1	156,992	123	49	71.9	11	0
Autographa californica MNPV	Baculoviridae	NC_001623	133,894	156	40.7	97.2	4	0
Cydia pomonella GV	Baculoviridae	NC_002816	123,500	143	45.3	90.1	2	0
Neodiprion setiferNPV	Baculoviridae	NC_005905	86,462	90	33.8	84.5	0	0
Spodoptera frugiperda GV	Baculoviridae	NC_026511.1	140,913	146	46.2	90.4	11	1
Trichoplusia ni GV LBIV-12	Baculoviridae	NC_038375.1	175,360	172	39.8	85.2	19	1
Glossina pallidipes SGHV	Hytrosaviridae	NC_010356	190,032	160	28	86.5	17	5

Musca domestica SGHV	Hytrosaviridae	EU522111	124,279	108	43.5	90.9	11	1
Oryctes rhinoceros NV	Nudiviridae	EU747721	127,615	139	41.6	88.5	1	0
Penaeus monodon NV	Nudiviridae	KJ184318	119,638	115	34.5	95.6	1	0
Tipula oleracea NV	Nudiviridae	KM610234	145,704	131	25.5	85.7	4	0
Apis mellifera FV	unassigned	KR819915	496,396	247	50.8	65	4	0
Drosophila-associated FV	unassigned	Multiple fragments, incomplete	86,478	69	33.5	Not reported	24	15
Leptopilina boulardi FV	unassigned	KY009685	111,453	108	21.3	80	33	23
M. hyperodae filamentous virus (MhFV)	unassigned	OQ439926	163,432	158	37.8	83	-	-

Supplementary Table 3 BlastP and HMMscan annotations for MhFV ORFs.

MhFV Gene ID	Start (bp)	End (bp)	Strand	Subject accession ID	Seq ID (%)	E-value	Species	BlastP hit kingdom	BlastP annotation	Pfam hit description	Pfam hit accession	Pfam query start	Pfam query end	Pfam domain i-evalue	Pfam domain c-evalue
ORF1	68	1492	-	YP_009116982.1	47.41	1.45E-125	Pseudoplusia includens SNPV IE	Viral	baculovirus repeat ORF	BRO family, N-terminal domain	PF02498.17	17	113	2.20E-24	6.10E-28
ORF2	2513	3115	+	YP_009165707.1	29.85	2.04E-11	Perigonia lusca single nucleopolyhedrovirus	Viral	Per os infectivity factor 3	Per os infectivity factor 3	PF05006.12	52	191	1.90E-11	3.20E-15
ORF3	3096	3512	-												
ORF4	3542	6229	+	YP_009345662.1	40.29	0	Leptopilina boulardi filamentous virus	Viral	putative DNA pol	DNA polymerase family B	PF00136.21	638	860	2.10E-15	2.30E-19
ORF5	6250	7017	+	QKN22518.1	38.16	3.82E-28	Drosophila-associated filamentous virus	Viral	putative DNA PolB, partial						
ORF6	7010	7366	+	YP_009345662.1	42.86	7.32E-15	Leptopilina boulardi	Viral	putative DNA pol						

							filamentous virus								
ORF7	7358	7765	-												
ORF8	7777	8034	-												
ORF9	8191	8379	-												
ORF10	8393	9010	-												
ORF11	9048	10961	-	QKN22485.1	29.39	3.04E-67	Drosophila-associated filamentous virus	Viral	putative ORF5						
ORF12	11129	11473	+	YP_009552615.1	47.87	1.26E-15	Operophtera brumata nucleopolyhedrovirus	Viral	BRO-A	BRO family, N-terminal domain	PF02498.17	16	86	2.00E-11	2.20E-15
ORF13	11517	13373	+	CAF4807074.1	39.32	4.92E-91	Pieris macdunnoughi	Eukaryote	unnamed protein product	Putative transposase DNA-binding domain	PF07282.11	525	586	1.50E-07	1.70E-11
ORF14	13393	14217	+	AOL57213.1	43.50	2.60E-50	Chrysodeixis includens nucleopolyhedrovirus	Viral	BRO-B	BRO family, N-terminal domain	PF02498.17	2	28	8.50E-05	9.50E-09
ORF15	14227	15429	+												
ORF16	15616	16359	-	AXU41742.1	33.16	1.52E-21	Spodoptera eridania nucleopolyhedrovirus	Viral	BRO-B	BRO family, N-terminal domain<br>T5orf172 domain<br>Meiotically up-regulated gene 113	PF02498.17<br>PF10544.9<br>PF13455.6	38<br>156<br>172	132<br>240<br>238	2.00E-17<br>4.00E-11<br>1.60E-10	4.40E-21<br>9.00E-15<br>3.50E-14
ORF17	16382	16930	-												
ORF18	16894	18048	-	AMB48743.1	28.08	3.28E-09	Glossina pallidipes salivary gland hypertrophy virus	Viral	Cg30-like protein						
ORF19	18911	19279	-												

ORF20	19293	21671	-	YP_009506121.1	27.98	3.71E-12	Trichoplusia ni granulovirus LBIV-12	Viral	hypothetical protein						
ORF21	21756	23999	+	NDE15715.1	43.86	5.04E-47	bacterium	Bacteria	hypothetical protein						
ORF22	24280	24852	-	YP_001649115.1	53.19	6.45E-19	Helicoverpa armigera granulovirus	Viral	BRO-H						
ORF23	24857	25303	-	YP_003517810.1	50.71	1.13E-40	Lymantria xylina nucleopolyhedrovirus	Viral	BRO-F	BRO family, N-terminal domain	PF02498.17	18	119	5.80E-27	3.20E-31
ORF24	25966	26532	+	XP_019754016.1	49.20	4.98E-61	Dendroctonus ponderosae	Eukaryote	PREDICTED: guanylate kinase isoform X3	Guanylate kinase<br>AAA domain<br>AAA domain<br>AAA domain<br>50S ribosome-binding GTPase<br>RsgA GTPase	PF00625.21<br>PF13671.6<br>PF13238.6<br>PF13401.6<br>PF01926.23<br>PF03193.16	1<br>5<br>6<br>4<br>5<br>4	182<br>143<br>143<br>30<br>59<br>25	5.30E-54<br>3.80E-07<br>7.10E-07<br>6.10E-05<br>3.70E-05<br>4.70E-05	7.40E-57<br>5.30E-10<br>9.90E-10<br>8.40E-08<br>5.20E-08<br>6.50E-08
ORF25	26525	26626	+												
ORF26	26789	27481	-	QEE79907.1	37.39	1.41E-14	Mamestra configurata nucleopolyhedrovirus A	Viral	Maco-A orf 20						
ORF27	27565	27693	-												
ORF28	28443	29462	-	WP_114909516.1	26.00	7.36E-06	Haemophilus haemolyticus	Eukaryote	autotransporter outer membrane beta-barrel domain-containing protein	Ezrin/radixin/moesin family	PF00769.19	39	139	7.00E-07	4.30E-10

ORF29	29488	30441	-	XP_025205964.1	31.92	4.29E-34	Melanaphis sacchari	Eukaryote	uncharacterized protein LOC112602222	KilA-N domain KilA-N domain	PF04383.13 PF04383.13	37 169	127 255	1.20E-12 8.70E-10	1.30E-16 9.70E-14
ORF30	30822	31364	-												
ORF31	32320	37275	+	YP_009345696.1	30.05	0	Leptopilina boulardi filamentous virus	Viral	hypothetical protein LbFV_ORF92						
ORF32	37844	38446	+												
ORF33	38458	39135	+												
ORF34	39135	39500	+												
ORF35	39530	39805	+	XP_028141650.1	42.68	3.94E-13	Diabrotica virgifera virgifera	Eukaryote	putative inhibitor of apoptosis isoform X1	Inhibitor of Apoptosis domain	PF00653.21	10	72	1.20E-16	2.60E-20
ORF36	39798	40499	+	YP_009345698.1	27.22	6.73E-08	Leptopilina boulardi filamentous virus	Viral	hypothetical protein LbFV_ORF94						
ORF37	40430	40594	-												
ORF38	41190	42071	+	YP_009116982.1	45.55	1.01E-82	Pseudoplusia includens SNPV IE	Viral	baculovirus repeat ORF	BRO family, N-terminal domain	PF02498.17	15	112	5.20E-24	1.20E-27
ORF39	42280	42540	+	YP_009121838.1	54.55	2.98E-18	Spodoptera frugiperda granulovirus	Viral	bro						
ORF40	42556	44613	-	YP_009345667.1	38.51	4.51E-135	Leptopilina boulardi filamentous virus	Viral	hypothetical protein LbFV_ORF63	Baculoviridae p74 conserved region Baculoviridae P74 N-terminal	PF04583.12 PF08404.10	384 12	601 295	5.80E-17 3.50E-15	6.50E-21 3.90E-19
ORF41	44761	45225	+	YP_762434.1	49.58	5.97E-30	Spodoptera frugiperda ascovirus 1a	Viral	40.2 kDa BRO-like protein	BRO family, N-terminal domain	PF02498.17	15	110	3.30E-25	1.80E-29
ORF42	45281	45595	+							Meiotically up-regulated gene 113	PF13455.6	10	45	0.00031	3.40E-08

ORF43	45629	46084	-												
ORF44	46119	47171	+												
ORF45	47168	47548	+												
ORF46	47519	47689	-												
ORF47	47863	49749	-	CAF4807074.1	38.90	2.61E-91	Pieris macdunnoughi	Eukaryote	unnamed protein product	Putative transposase DNA-binding domain	PF07282.11	535	596	1.60E-07	1.70E-11
ORF48	49800	50321	-	MBR3706763.1	56.92	7.00E-13	Firmicutes bacterium	Bacteria	Bro-N domain-containing protein	BRO family, N-terminal domain	PF02498.17	1	22	0.00019	3.20E-08
ORF49	50392	50598	-	YP_003517810.1	50.79	6.53E-11	Lymantria xylina nucleopolyhedrovirus	Viral	BRO-F	BRO family, N-terminal domain	PF02498.17	16	61	1.60E-09	8.90E-14
ORF50	50698	51744	-	YP_003517810.1	34.53	1.31E-17	Lymantria xylina nucleopolyhedrovirus	Viral	BRO-F	BRO family, N-terminal domain	PF02498.17	37	133	1.70E-19	2.80E-23
ORF51	51767	52399	-	XP_019852807.1	34.78	3.23E-05	Amphimedon queenslandica	Eukaryote	PREDICTED: uncharacterized protein LOC100632541						
ORF52	52363	53577	-	GBO32095.1	28.50	1.35E-08	Araneus ventricosus	Eukaryote	hypothetical protein AVEN_250922-1						
ORF53	54715	55014	-												
ORF54	55084	56448	-	YP_009116982.1	44.22	4.57E-136	Pseudoplusia includens SNPV IE	Viral	baculovirus repeat ORF	BRO family, N-terminal domain	PF02498.17	15	110	7.50E-25	1.30E-28
ORF55	56599	57000	-	YP_001883364.1	33.08	7.01E-11	Musca domestica salivary gland hypertrophy virus	Viral	matrix metalloproteinase	Matrixin	PF00413.24	26	127	9.50E-13	2.10E-16
ORF56	57113	57733	+												
ORF57	57787	59799	-	YP_001649041.1	40.37	1.02E-12	Helicoverpa armigera granulovirus	Viral	hypothetical protein HaGV_gp059						

ORF58	59893	61149	+	NDE18639.1	29.07	1.89E-20	bacterium	Bacteria	hypothetical protein					
ORF59	61176	61607	+	ADY88154.1	54.55	4.04E-46	Helicoverpa armigera SNPV	Viral	BRO-B, partial	BRO family, N-terminal domain	PF02498.17	15	111	7.00E-25	7.90E-29
ORF60	62575	64689	-											
ORF61	64718	65056	-											
ORF62	65061	67538	-											
ORF63	67528	68580	-	YP_009345647.1	26.46	1.09E-16	Leptopilina boulardi filamentous virus	Viral	hypothetical protein LbFV_ORF43					
ORF64	68599	69528	+	QKN22505.1	46.37	1.66E-82	Drosophila-associated filamentous virus	Viral	putative protein 51					
ORF65	69743	70933	+											
ORF66	71062	71487	-											
ORF67	71627	73516	-	YP_009345617.1	28.70	1.17E-23	Leptopilina boulardi filamentous virus	Viral	JmJC domain protein	JmjC domain, hydroxylase	PF02373.22	331	449	4.20E-15	2.30E-18
ORF68	73777	74229	+											
ORF69	74076	74489	-											
ORF70	74628	75218	+											
ORF71	75366	76313	-	YP_009345656.1	31.05	3.18E-30	Leptopilina boulardi filamentous virus	Viral	hypothetical protein LbFV_ORF52					
ORF72	76870	78018	-	YP_001649139.1	32.20	8.89E-56	Helicoverpa armigera granulovirus	Viral	hypothetical protein HaGV_gp157					
ORF73	78165	79802	-	GBO32095.1	22.77	3.24E-21	Araneus ventricosus	Eukaryote	hypothetical protein AVEN_250922-1					
ORF74	81133	82167	+	QKN22504.1	28.95	3.24E-47	Drosophila-associated filamentous virus	Viral	putative lecithine cholesterol acyltransferase					

ORF75	82177	82485	+												
ORF76	82649	82834	+												
ORF77	82849	83100	+												
ORF78	83138	83809	+	YP_009345689.1	34.42	3.58E-20	Leptopilina boulardi filamentous virus	Viral	hypothetical protein LbFV_ORF85	Baculoviridae AC81	PF05820.11	53	206	1.30E-21	7.30E-26
ORF79	83855	84367	+	XP_029617680.1	41.67	5.14E-10	Salmo trutta	Eukaryote	F-box only protein 41-like						
ORF80	84408	84749	+	YP_009345691.1	40.20	3.44E-10	Leptopilina boulardi filamentous virus	Viral	hypothetical protein LbFV_ORF87						
ORF81	84715	84954	+												
ORF82	84951	85700	+												
ORF83	85693	86448	+	YP_009345623.1	40.11	6.24E-29	Leptopilina boulardi filamentous virus	Viral	hypothetical protein LbFV_ORF19	Protein of unknown function (DUF705)<br>NLI interacting factor-like phosphatase	PF05152.12<br>PF03031.18	57<br>79	143<br>211	3.10E-09<br>1.80E-07	1.20E-12<br>7.10E-11
ORF84	86460	86711	-												
ORF85	86723	87439	-												
ORF86	87751	88077	-												
ORF87	88703	89617	-												
ORF88	89676	89861	-												
ORF89	89876	90871	-	QKN22457.1	30.59	1.28E-36	Drosophila-associated filamentous virus	Viral	putative ORF2	Phage integrase family	PF00589.22	152	311	3.80E-12	4.30E-16
ORF90	90941	92800	-	WP_142941407.1	26.51	7.39E-37	Aliikangiella marina	Bacteria	hypothetical protein						
ORF91	92872	94449	-	QKN22521.1	27.73	2.63E-18	Drosophila-associated filamentous virus	Viral	putative ORF107						

ORF92	94603	96675	+	YP_009345710.1	38.78	2.36E-151	Leptopilina boulardi filamentous virus	Viral	putative ODV protein	Polysaccharide lyase family 8, N terminal alpha-helical domain Baculovirus E66 occlusion-derived virus envelope protein	PF08124.11 PF04850.12	85 355	271 486	1.60E-11 1.90E-06	2.60E-15 3.20E-10
ORF93	96595	97122	-												
ORF94	97119	97238	-												
ORF95	97661	97834	+												
ORF96	97850	98641	-	XP_038668136.1	25.13	4.81E-14	Scyliorhinus canicula	Eukaryote	BICD family-like cargo adapter 2						
ORF97	98664	100463	+	QKN22506.1	24.75	1.64E-23	Drosophila-associated filamentous virus	Viral	PIF1-like protein	Per os infectivity	PF05092.12	64	450	1.40E-08	1.60E-12
ORF98	101147	101344	+												
ORF99	101567	102769	+	AMB48743.1	26.71	1.37E-07	Glossina pallidipes salivary gland hypertrophy virus	Viral	Cg30-like protein						
ORF100	102733	103320	+												
ORF101	103349	104467	-												
ORF102	104745	106253	-												
ORF103	106266	106466	-												
ORF104	106463	106906	-												
ORF105	107682	109649	-	XP_025205964.1	26.23	5.13E-44	Melanaphis sacchari	Eukaryote	uncharacterized protein LOC112602222	KilA-N domain KilA-N domain Helicobacter pylori protein of unknown	PF04383.13 PF04383.13 PF05917.11 PF00769.19	30 162 341 351	115 243 418 453	5.90E-12 8.10E-07	3.60E-15 5.00E-10 7.30E-

										function (DUF874)<br>Ezrin/radixin/moesin family				0.00012<br>0.00016	08<br>9.90E-08
ORF106	110286	111314	-	VVC35478.1	38.19	1.12E-14	Cinara cedri	Eukaryote	Hypothetical protein CINCED_3A011688						
ORF107	111404	112270	-	XP_025205964.1	32.25	2.61E-34	Melanaphis sacchari	Eukaryote	uncharacterized protein LOC112602222	KilA-N domain<br>KilA-N domain	PF04383.13<br>PF04383.13	29<br>158	116<br>244	7.50E-12<br>6.30E-08	4.20E-16<br>3.50E-12
ORF108	112922	113362	+												
ORF109	113348	113920	-												
ORF110	114674	114823	-												
ORF111	115605	116087	+	YP_009345628.1	30.83	1.57E-06	Leptopilina boulardi filamentous virus	Viral	hypothetical protein LbFV_ORF24						
ORF112	116084	119035	+	YP_009345700.1	37.84	0	Leptopilina boulardi filamentous virus	Viral	hypothetical protein LbFV_ORF96						
ORF113	119157	121202	-	XP_025205964.1	25.31	1.12E-42	Melanaphis sacchari	Eukaryote	uncharacterized protein LOC112602222	KilA-N domain<br>KilA-N domain	PF04383.13<br>PF04383.13	30<br>159	120<br>244	1.10E-12<br>5.20E-08	5.10E-16<br>2.30E-11
ORF114	121403	121636	-												
ORF115	122122	122778	-	AXU41742.1	30.94	3.65E-08	Spodoptera eridania nucleopolyhedrovirus	Viral	BRO-B	Meiotically up-regulated gene 113<br>T5orf172 domain	PF13455.6<br>PF10544.9	138<br>124	207<br>201	1.60E-11<br>4.10E-10	2.70E-15<br>6.80E-14
ORF116	122836	123867	+	WP_052044918.1	32.12	2.20E-30	Arsenophonus endosymbiont	Bacteria	lytic polysaccharide monooxygenase	Lytic polysaccharide mono-	PF03067.15	34	205	4.60E-09	5.10E-13

							of Nilaparvata lugens			oxygenase, cellulose-degrading
ORF117	123864	124799	+							
ORF118	124800	125627	-							
ORF119	125629	126402	+							
ORF120	126447	128117	+							
ORF121	128169	128321	+							
ORF122	128361	128813	-							
ORF123	129362	130327	-	YP_009345706.1	29.23	9.25E-30	Leptopilina boulardi filamentous virus	Viral	hypothetical protein LbFV_ORF102	
ORF124	130443	131729	+	YP_009345687.1	24.54	4.74E-13	Leptopilina boulardi filamentous virus	Viral	hypothetical protein LbFV_ORF83	
ORF125	131917	132492	+							
ORF126	132664	133368	+	YP_009345641.1	33.48	1.87E-22	Leptopilina boulardi filamentous virus	Viral	nudix domain protein	
ORF127	133368	134324	+	QKN22511.1	37.59	2.28E-17	Drosophila-associated filamentous virus	Viral	putative protein 57	
ORF128	134275	135486	+	YP_009345624.1	33.52	7.01E-52	Leptopilina boulardi filamentous virus	Viral	hypothetical protein LbFV_ORF20	
ORF129	135561	135857	-							

ORF130	135883	136680	-	YP_001686976.1	47.37	1.75E-11	Glossina pallidipes salivary gland hypertrophy virus	Viral	hypothetical protein SGHV028	MSV199 domain	PF10553.9	43	106	7.60E-10	4.20E-13
ORF131	136868	137305	+												
ORF132	137349	138380	+	YP_009345671.1	36.52	8.95E-52	Leptopilina boulardi filamentous virus	Viral	hypothetical protein LbFV_ORF67						
ORF133	138383	139012	+	WP_209862961.1	28.43	3.39E-10	Paenibacillus shirakamiensis	Bacteria	lytic polysaccharide monooxygenase	Lytic polysaccharide mono-oxygenase, cellulose-degrading	PF03067.15	15	201	8.20E-07	4.60E-11
ORF134	139367	139846	-												
ORF135	139873	140460	-	AMB48743.1	28.08	1.11E-09	Glossina pallidipes salivary gland hypertrophy virus	Viral	Cg30-like protein						
ORF136	140408	141028	-							Drought induced 19 protein (Di19), zinc-binding	PF05605.12	16	54	0.00031	3.40E-08
ORF137	141392	141571	-												
ORF138	141812	142609	-												
ORF139	142892	144097	+	NDE18639.1	29.07	6.69E-21	bacterium	Bacteria	hypothetical protein						
ORF140	144112	144258	-												
ORF141	144263	146206	-	YP_009345682.1	40.82	9.85E-146	Leptopilina boulardi filamentous virus	Viral	hypothetical protein LbFV_ORF78						

ORF142	146494	147312	-	YP_009345703.1	30.95	8.66E-06	Leptopilina boulardi filamentous virus	Viral	hypothetical protein LbFV_ORF99						
ORF143	147951	148226	-												
ORF144	148306	149508	-	YP_009345685.1	38.95	4.25E-62	Leptopilina boulardi filamentous virus	Viral	putative ATPase	ATPase family associated with various cellular activities (AAA)<br>AAA ATPase domain<br>Protein of unknown function (DUF815)<br>Holliday junction DNA helicase RuvB P-loop domain<br>AAA domain (dynein-related subfamily)<br>AAA domain<br>ABC transporter<br>AAA domain (Cdc48 subfamily)<br>TIP49 P-loop domain	PF00004.29<br>PF13191.6<br>PF05673.13<br>PF05496.12<br>PF07728.14<br>PF13173.6<br>PF00005.27<br>PF07724.14<br>PF06068.13	198<br>172<br>172<br>168<br>198<br>196<br>187<br>197<br>170	325<br>270<br>320<br>319<br>309<br>279<br>257<br>271<br>247	3.80E-23<br>2.30E-08<br>4.90E-08<br>2.70E-06<br>7.80E-06<br>2.70E-05<br>2.10E-05<br>0.00033<br>0.00047	8.00E-26<br>4.80E-11<br>1.00E-10<br>5.60E-09<br>1.70E-08<br>5.60E-08<br>4.30E-08<br>7.00E-07<br>1.00E-06
ORF145	149514	149843	-												
ORF146	149921	150667	-												
ORF147	150989	151135	-												
ORF148	151418	153529	+	YP_009345672.1	32.07	2.29E-103	Leptopilina boulardi filamentous virus	Viral	hypothetical protein LbFV_ORF68	ATPase domain predominantly from Archaea<br>AAA domain<br>AAA domain	PF01637.18<br>PF13604.6<br>PF13245.6	177<br>169<br>191	336<br>359<br>345	5.00E-06<br>6.40E-05	5.30E-09<br>6.70E-08

														3.20E-05	3.40E-08
ORF149	153576	153839	+												
ORF150	153894	154406	-	YP_001687091.1	28.69	5.46E-08	Glossina pallidipes salivary gland hypertrophy virus	Viral	hypothetical protein SGHV143						
ORF151	154486	155229	-	YP_009049844.1	29.83	1.74E-23	Peridroma alphabaculovirus	Viral	bro	BRO family, N-terminal domain<br>Meiotically up-regulated gene 113<br>T5orf172 domain	PF02498.17<br>PF13455.6<br>PF10544.9	38<br>172<br>156	132<br>239<br>236	3.00E-19<br>9.30E-12<br>5.70E-11	5.00E-23<br>1.60E-15<br>9.60E-15
ORF152	155253	156047	-	XP_023022306.1	48.04	2.19E-89	Leptinotarsa decemlineata	Eukaryote	baculoviral IAP repeat-containing protein 2	Inhibitor of Apoptosis domain<br>Inhibitor of Apoptosis domain<br>Zinc finger, C3HC4 type (RING finger)	PF00653.21<br>PF00653.21<br>PF13920.6	9<br>113<br>216	73<br>178<br>257	5.20E-16<br>2.40E-22<br>1.40E-09	1.20E-19<br>5.40E-26<br>3.10E-13
ORF153	156153	156527	-												
ORF154	156549	157553	-	WP_104124158.1	30.20	1.99E-14	Acinetobacter soli	Bacteria	Bro-N domain-containing protein	BRO family, N-terminal domain	PF02498.17	38	133	2.70E-17	3.00E-21
ORF155	157588	159219	-	GBO25838.1	24.76	3.93E-18	Araneus ventricosus	Eukaryote	hypothetical protein AVEN_65616-1						
ORF156	160095	161300	-	MBO1045201.1	39.09	3.20E-05	Aphanizomenon flos-aquae	Eukaryote	Uma2 family endonuclease						
ORF157	161458	162342	-	XP_025205964.1	30.87	3.68E-32	Melanaphis sacchari	Eukaryote	uncharacterized protein LOC112602222	KilA-N domain<br>KilA-N domain	PF04383.13<br>PF04383.13	26<br>153	114<br>244	2.00E-11	1.10E-15

				3.00E-09	1.70E-13
ORF158	162611	162763	+		

Supplementary Table 4 Significant differentially expressed genes (DEGs) from DESeq2 MhFV tissue LRT analysis.

MhFV differentially expressed gene ID	DESeq2 baseMean	DESeq2 log2FoldChange	DESeq2 lfcSE	DESeq2 LRT stat	DESeq2 padj
ORF67	5.64	-1.03	2.07	20.88	6.70E-03
ORF57	11.70	2.37	1.37	17.48	1.04E-02
ORF156	10.76	-1.68	1.89	18.34	1.04E-02
ORF141	3.31	1.31	2.47	16.02	1.50E-02
ORF96	3.88	0.90	2.73	15.27	1.67E-02
ORF125	3.80	3.52	1.91	13.08	3.63E-02
ORF126	3.43	4.49	2.09	12.23	4.49E-02
ORF47	3.91	2.72	2.74	11.26	4.65E-02
ORF54	2.70	3.12	2.60	11.31	4.65E-02
ORF105	2.21	1.18	2.68	10.89	4.65E-02
ORF113	2.40	2.94	2.80	10.89	4.65E-02
ORF116	4.20	-0.81	2.43	10.97	4.65E-02

Supplementary Table 5 Accessions for genomes used for *Microctonus* species divergence estimate analysis.

Organism	Code	NCBI Accession
Atta cephalotes	acep	PRJNA279976
Apis cerana	acer	PRJNA324433
Acromyrmex echinatior	aech	PRJNA271903
Apis mellifera	amel	PRJNA477511
Bombus impatiens	bimp	PRJNA70395
Bombus terrestris	bter	PRJNA68545
Cerapachys biroi	cbir	PRJNA275884
Ceratina calcarata	ccal	PRJNA340002
Camponotus floridanus	cflo	PRJNA476946
Cardiocondyla obscurior	cobs	Cobs_1.4 assembly accessed from https://hymenoptera.elsiklab.missouri.edu/ant_genomes/cardiocondyla
Drosophila melanogaster	dmel	PRJNA13812
Dufourea novaeangliae	dnov	PRJNA311229
Formica exsecta	fexs	PRJNA552586
Harpegnathos saltator	hsal	PRJNA273397
Linepithema humile	lhum	PRJNA281950
Monomorium pharaonis	mpha	PRJNA285339
Megachile rotundata	mrot	PRJNA87021
Nasonia vitripennis	nvit	PRJNA20073

Pogonomyrmex barbatus	pbar	PRJNA276107
Solenopsis invicta	sinv	PRJNA268798
Trichogramma pretiosum	tpre	PRJNA297592
Vollenhovia emeryi	veme	PRJNA278668
Vespula germanica	vger	PRJNA643352
Vespula pensylvanica	vpen	PRJNA643352
Vespula vulgaris	vvul	PRJNA643352
Wasmannia auropunctata	waur	PRJNA279179

Supplementary Table 6 Accessions for virus genes used to construct nuclear arthropod-specific large double-stranded DNA virus phylogeny.

Gene	Organism	Protein accession number (NCBI, Ag Data Commons or Locus Tag)
ac81	AcMNPV	NP_054111.1
DNA polymerase	AcMNPV	NP_054095.1
helicase	AcMNPV	NP_054125.1
lef-5	AcMNPV	NP_054129.1
lef-8	AcMNPV	NP_054079.1
lef-9	AcMNPV	NP_054092.1
p33	AcMNPV	NP_054122.1
pif-0	AcMNPV	NP_054168.1
pif-1	AcMNPV	NP_054149.1
pif-2	AcMNPV	NP_054051.1
pif-3	AcMNPV	NP_054145.1
pif-5	AcMNPV	NP_054179.1
ac81	AmFV	YP_009165894.1
DNA polymerase	AmFV	YP_009165825.1
lef-5	AmFV	YP_009165903.1
pif-0	AmFV	YP_009165828.1
pif-1	AmFV	YP_009165811.1
pif-2	AmFV	YP_009165851.1
pif-3	AmFV	YP_009165839.1
pif-5	AmFV	YP_009165808.1
DNA polymerase	CoBV	GAV93177.1
pif-0	CoBV	GAV93149.1
pif-1	CoBV	GAV93135.1
pif-2	CoBV	GAV93160.1
pif-3	CoBV	GAV93232.1
pif-5	CoBV	GAV93153.1
ac81	CpGV	NP_148887.1
DNA polymerase	CpGV	NP_148895.1
helicase	CpGV	NP_148874.1
lef-5	CpGV	NP_148871.1
lef-8	CpGV	NP_148915.1
lef-9	CpGV	NP_148901.1
p33	CpGV	NP_148877.1
pif-0	CpGV	NP_148844.1
pif-1	CpGV	NP_148859.1
pif-2	CpGV	NP_148832.1
pif-3	CpGV	NP_148819.1

pif-5	CpGV	NP_148802.1
ac81	CuniNPV	NP_203410.1
DNA polymerase	CuniNPV	NP_203396.1
helicase	CuniNPV	NP_203394.1
lef-5	CuniNPV	NP_203391.1
lef-8	CuniNPV	NP_203330.1
lef-9	CuniNPV	NP_203363.1
p33	CuniNPV	NP_203317.1
pif-0	CuniNPV	NP_203378.1
pif-1	CuniNPV	NP_203333.1
pif-2	CuniNPV	NP_203342.1
pif-3	CuniNPV	NP_203350.1
pif-5	CuniNPV	NP_203406.1
ac81	DaFV	QKN22513.1
DNA polymerase	DaFV	QKN22518.1
helicase	DaFV	-
lef-5	DaFV	-
lef-8	DaFV	QKN22516.1
lef-9	DaFV	QKN22481.1
p33	DaFV	QKN22511.1
pif-0	DaFV	QKN22456.1
pif-1	DaFV	QKN22506.1
pif-2	DaFV	QKN22478.1
pif-3	DaFV	-
pif-5	DaFV	QKN22500.1
ac81	DiNV	ATZ81543.1
DNA polymerase	DiNV	YP_009553129.1
helicase	DiNV	YP_009553076.1
lef-5	DiNV	YP_009553089.1
lef-8	DiNV	YP_009553096.1
lef-9	DiNV	YP_009553121.1
p33	DiNV	YP_009553151.1
pif-0	DiNV	ATZ81555.1
pif-1	DiNV	ATZ81516.1
pif-2	DiNV	ATZ81582.1
pif-3	DiNV	ATZ81572.1
pif-5	DiNV	ATZ81566.1
ac81	GbNV	YP_001111281.1
DNA polymerase	GbNV	YP_001111279.1
helicase	GbNV	YP_001111355.1
lef-5	GbNV	YP_001111352.1

lef-8	GbNV	YP_001111316.1
lef-9	GbNV	YP_001111291.1
p33	GbNV	YP_001111274.1
pif-0	GbNV	YP_001111312.1
pif-1	GbNV	YP_001111319.1
pif-2	GbNV	YP_001111333.1
pif-3	GbNV	YP_001111270.1
pif-5	GbNV	YP_001111272.1
ac81	GpSGHV	YP_001687026.1
DNA polymerase	GpSGHV	YP_001687027.1
helicase	GpSGHV	YP_001686993.1
lef-8	GpSGHV	YP_001686988.1
lef-9	GpSGHV	YP_001686981.1
p33	GpSGHV	YP_001687020.1
pif-0	GpSGHV	YP_001686949.1
pif-1	GpSGHV	YP_001687050.1
pif-2	GpSGHV	YP_001687001.1
pif-3	GpSGHV	YP_001687024.1
pif-5	GpSGHV	YP_001687044.1
ac81	HzNV-2	YP_004956844.1
DNA polymerase	HzNV-2	YP_004956766.1
helicase	HzNV-2	YP_004956786.1
lef-5	HzNV-2	YP_004956788.1
lef-8	HzNV-2	YP_004956799.1
lef-9	HzNV-2	YP_004956811.1
pif-0	HzNV-2	YP_004956854.1
pif-1	HzNV-2	YP_004956830.1
pif-2	HzNV-2	YP_004956774.1
pif-3	HzNV-2	YP_004956801.1
pif-5	HzNV-2	YP_004956810.1
ac81	LbFV	YP_009345689.1
DNA polymerase	LbFV	YP_009345662.1
helicase	LbFV	YP_009345671.1
lef-8	LbFV	YP_009345700.1
lef-9	LbFV	YP_009345682.1
p33	LbFV	YP_009345701.1
pif-0	LbFV	YP_009345667.1
pif-1	LbFV	YP_009345636.1
pif-2	LbFV	YP_009345696.1
pif-5	LbFV	YP_009345647.1
ac81	MdSGHV	YP_001883436.1

DNA polymerase	MdSGHV	YP_001883329.1
lef-8	MdSGHV	YP_001883398.1
lef-9	MdSGHV	YP_001883402.1
p33	MdSGHV	YP_001883430.1
pif-0	MdSGHV	YP_001883367.1
pif-1	MdSGHV	YP_001883357.1
pif-2	MdSGHV	YP_001883417.1
pif-3	MdSGHV	YP_001883434.1
pif-5	MdSGHV	YP_001883353.1
ac81	MhFV	MhFV_ORF78
DNA polymerase	MhFV	MhFV_ORF4
helicase	MhFV	MhFV_ORF132
lef-5	MhFV	-
lef-8	MhFV	MhFV_ORF112
lef-9	MhFV	MhFV_ORF141
p33	MhFV	MhFV_ORF127
pif-0	MhFV	MhFV_ORF40
pif-1	MhFV	MhFV_ORF97
pif-2	MhFV	MhFV_ORF31
pif-3	MhFV	MhFV_ORF2
pif-5	MhFV	MhFV_ORF63
ac81	NeseNPV	YP_025155.1
DNA polymerase	NeseNPV	YP_025135.1
helicase	NeseNPV	YP_025168.1
lef-5	NeseNPV	YP_025165.1
lef-8	NeseNPV	YP_025188.1
lef-9	NeseNPV	YP_025147.1
p33	NeseNPV	YP_025131.1
pif-0	NeseNPV	YP_025157.1
pif-1	NeseNPV	YP_025186.1
pif-2	NeseNPV	YP_025162.1
pif-3	NeseNPV	YP_025176.1
pif-5	NeseNPV	YP_025145.1
ac81	OrNV	YP_002321315.1
DNA polymerase	OrNV	YP_002321312.1
helicase	OrNV	YP_002321345.1
lef-5	OrNV	YP_002321363.1
lef-8	OrNV	YP_002321375.1
lef-9	OrNV	YP_002321407.1
p33	OrNV	YP_002321424.1
pif-0	OrNV	YP_002321437.1

pif-1	OrNV	YP_002321371.1
pif-2	OrNV	YP_002321328.1
pif-3	OrNV	YP_002321418.1
pif-5	OrNV	YP_002321426.1
ac81	PmNV	YP_009051924.1
DNA polymerase	PmNV	YP_009051843.1
helicase	PmNV	YP_009051932.1
lef-5	PmNV	YP_009051890.1
lef-8	PmNV	YP_009051861.1
lef-9	PmNV	YP_009051896.1
p33	PmNV	YP_009051846.1
pif-0	PmNV	YP_009051910.1
pif-1	PmNV	YP_009051877.1
pif-2	PmNV	YP_009051853.1
pif-3	PmNV	YP_009051931.1
pif-5	PmNV	YP_009051848.1
ac81	ToNV	YP_009116770.1
DNA polymerase	ToNV	YP_009116659.1
helicase	ToNV	YP_009116765.1
lef-5	ToNV	YP_009116697.1
lef-8	ToNV	YP_009116735.1
lef-9	ToNV	YP_009116778.1
p33	ToNV	YP_009116746.1
pif-0	ToNV	YP_009116692.1
pif-1	ToNV	YP_009116716.1
pif-2	ToNV	YP_009116654.1
pif-3	ToNV	YP_009116660.1
pif-5	ToNV	YP_009116721.1
DNA polymerase	WSSV	YP_009220649.1
pif-0	WSSV	YP_009220510.1
pif-1	WSSV	YP_009220545.1
pif-2	WSSV	YP_009220486.1
pif-3	WSSV	YP_009220581.1
pif-5	WSSV	YP_009220481.1
